# Plasma androgen receptor and serum chromogranin A in advanced prostate cancer

**DOI:** 10.1038/s41598-018-33774-4

**Published:** 2018-10-18

**Authors:** Vincenza Conteduca, Emanuela Scarpi, Samanta Salvi, Valentina Casadio, Cristian Lolli, Giorgia Gurioli, Giuseppe Schepisi, Daniel Wetterskog, Alberto Farolfi, Cecilia Menna, Delia De Lisi, Salvatore Luca Burgio, Himisha Beltran, Gerhardt Attard, Ugo De Giorgi

**Affiliations:** 1 Department of Medical Oncology, Istituto Scientifico Romagnolo per lo Studio e la Cura dei Tumori (IRST) IRCCS, via Maroncelli 40, 47014 Meldola, Italy; 2 The Institute of Cancer Research and the Royal Marsden, 15 Cotswold Road, Sutton, Surrey SM2 5NG UK; 3 Unit of Biostatistics and Clinical Trials, Istituto Scientifico Romagnolo per lo Studio e la Cura dei Tumori (IRST) IRCCS, via Maroncelli 40, 47014 Meldola, Italy; 4 Biosciences Laboratory, Istituto Scientifico Romagnolo per lo Studio e la Cura dei Tumori (IRST) IRCCS, via Maroncelli 40, 47014 Meldola, Italy; 5 Medical Oncology Department, Campus Bio-Medico University, Via Alvaro del Portillo 200, 00128 Rome, Italy; 6 Division of Medical Oncology, Weill Cornell Medicine, New York, NY 10021 USA; 7 Academic Urology Unit, The Royal Marsden NHS Foundation Trust, London, UK

## Abstract

Recently, mixed forms between adenocarcinoma and neuroendocrine prostate cancer (NEPC) have emerged that are characterized by persistent androgen receptor (AR)-signalling and elevated chromogranin A (CgA) levels. The main aim of this study was to analyze castration-resistant prostate cancer (CRPC) patients treated with abiraterone or enzalutamide, assessing progression-free/overall survival (PFS/OS) in association with circulating *AR* and CgA. *AR* aberrations were analyzed by droplet digital PCR in pre-treatment plasma samples collected from two biomarker protocols [197 patients from a retrospective study (REC 2192/2013) and 59 from a prospective trial (REC 6798/2015)]. We subdivided patients into three groups according to CgA by receiver-operating characteristic (ROC) curves. In the primary cohort, plasma *AR* gain and mutations (p.L702H/p.T878A) were detected in 78 (39.6%) and 16 (8.1%) patients, respectively. We observed a significantly worse PFS/OS in patients with higher-CgA than in patients with normal-CgA, especially those with no *AR*-aberrations. Multivariable analysis showed *AR* gain, higher-CgA and LDH levels as independent predictors of PFS [hazard ratio (HR) = 2.16, 95% confidence interval (95% CI) 1.50–3.12, p < 0.0001, HR = 1.73, 95% CI 1.06–2.84, p = 0.026, and HR = 2.13, 95% CI 1.45–3.13, p = 0.0001, respectively) and OS (HR = 1.72, 95% CI 1.15–2.57, p = 0.008, HR = 3.63, 95% CI 2.13–6.20, p < 0.0001, and HR = 2.31, 95% CI 1.54–3.48, p < 0.0001, respectively). These data were confirmed in the secondary cohort. Pre-treatment CgA detection could be useful to identify these mixed tumors and would seem to have a prognostic role, especially in *AR*-normal patients. This association needs further evaluation in larger prospective cohorts.

## Introduction

Prostate cancer is the most commonly diagnosed cancer in men worldwide and a major cause of cancer death^1^. After an initial response to medical or surgical castration, the disease will progress to castration-resistant prostate cancer (CRPC)^2^. Many drugs with different mechanisms of action are currently available for CRPC patients and have led to a significant benefit in survival^3–8^. However, the identification of predictive factors could help clinicians to better select the next-line systemic therapy on the basis of molecular risk stratification. The implementation of these biomarkers in routine clinical practice would substantially facilitate patient-tailored treatment.

The molecular landscape of CRPC provides insights into the mechanisms of tumor heterogeneity and treatment resistance^9–11^. Resistance in CRPC is typically driven by the reactivation of androgen receptor (AR) signalling, occuring through AR splice variants (such as AR-V7), and AR point mutations or amplification, which can be detected in blood^12–20^. In recent years, cell-free DNA has emerged as a promising and non-invasive biomarker for the characterization of the tumor genome^21^. Approximately 3% of tumor DNA is released into the blood daily, most likely deriving from apoptosis/necrosis of malignant cells^22^. High levels of cell-free DNA (4- to 6-fold that of healthy controls) have been detected in patients with tumors, including prostate cancer^22^. Recently, AR gene aberrations detected in cell-free DNA of CRPC patients using PCR-based methods or next generation sequencing have been associated with worse outcome and resistance to abiraterone and enzalutamide, suggesting a potential predictive/prognostic role for plasma *AR* status^12–17^.

In CRPC, neuroendocrine differentiation (NED) represents an alternative *AR*-independent mechanism of resistance to cancer therapeutics, especially androgen deprivation agents. Neuroendocrine prostate cancer (NEPC) is uncommon and characterized by decreased prostate specific antigen (PSA) secretion, and usually by the expression of neuroendocrine markers, such as chromogranin A (CgA), synaptophysin, and neuron specific enolase (NSE)^23,24^. In addition, NEPC usually shows loss of AR expression and suppressed AR signalling, while *AR* aberrations (point mutation and gain) are present at low levels, probably because of clonal selection of non-amplified prostate adenocarcinoma subpopulations through selective pressure, especially during anti-AR therapies^24^. *De novo* small-cell prostate cancer, a highly aggressive histologic variant, is present in <1% of untreated prostate cancers, whereas the frequency of treatment-related NEPC has been reported as occurring in up to 20% of patients during the course of CRPC progression^23–25^. A recent classification of neuroendocrine prostatic tumors^26^ showed variants of neuroendocrine prostate cancer, including a mixed form between NEPC and conventional adenocarcinoma, usually characterized by AR independence, However, mixed tumors have been also observed with both AR positive and AR negative cells and, on occasion, with dual expression of both neuroendocrine markers and AR in the same tumor cells due to inter- and intra-patient clinical and pathologic heterogeneity. Consequently, a more accurate clinical and molecular classification is needed for these overlapping clinical entities, and further research is warranted to identify their prognostic impact^27^.

In addition, elevated levels of serum CgA, commonly observed in NEPC, may increase in CRPC patients with adenocarcinoma histology^28^ who show a shorter survival than those with normal CgA values^29,30^.

Our main objective was to identify the correlation between *AR* status and CgA level before the administration of anti-AR therapies and in different settings (chemotherapy-naïve and chemotherapy-treated patients) in prostate adenocarcinoma. We also evaluated the impact on treatment outcome of CgA levels in association with cell-free *AR* status.

## Results

### Overall patient characteristics

The primary cohort included 197 patients from a retrospective biomarker study (REC 2192/2013) and the secondary cohort consisted of 59 from a prospective biomarker trial (REC 6798/2015). Patients with available pre-treatment serum CgA and plasma DNA for detection of AR gene aberration were considered evaluable. All patients in both cohorts underwent prostate biopsy and/or prostatectomy at diagnosis with a confirmed histology of prostate adenocarcinoma without NED. Median age was 73 years (range, 42–91) and 75 (range, 48–89) in the primary and secondary cohorts, respectively. The prospective biomarker trial was more recent than retrospective study and, consequently, included many more chemotherapy-naïve cases treated with abiraterone or enzalutamide (N = 38, 64.4%) than the retrospective study (N = 40, 20.3%). This substantial difference may justify the different baseline characteristics between the two cohorts (e.g., presence of visceral metastases and number of previous treatments).

The median serum CgA level was 122 ng/mL (range, 10–1000) (normal CgA value < 120 ng/mL). However, the receiver-operating characteristic (ROC) analysis, one of the most commonly used methods to analyze the effectiveness of a diagnostic, was used to evaluate the role of pre-treatment serum CgA for assessing the response to no response to treatment with abiraterone or enzalutamide. The cutoffs for CgA in response to AR-directed therapies were calculated through the area under the ROC curve (AUC), as previously reported^29,30^. In the primary cohort, serum CgA level was considered normal in 92 (46.7%) patients, elevated but ≤ 3-fold the upper normal value (UNV) in 66 (33.5%) and ≥3-fold the UNV in 39 (20.3%) patients. A similar distribution of CgA levels was observed in the secondary cohort. We assessed *AR* status in cell-free DNA showing *AR* copy number (CN) gain in 78 (39.6%) and *AR* mutations (p.L702H or p.T878A) in 16 (8.1%) patients of the primary cohort. In the secondary cohort, plasma *AR* gain and mutations were reported in 11 (18.6%) and 2 (3.4%) patients, respectively. All patient characteristics are summarized in Table 1.

**Table 1:** Patient characteristics in the Primary and Secondary Cohort.

	Primary Cohort			Secondary Cohort		
	Total (n = 197)	Abi/Enza pre-docetaxel (n = 40)	Abi/Enza post-docetaxel (n = 157)	Total (n = 59)	Abi/Enza pre-docetaxel (n = 38)	Abi/Enza post-docetaxel (n = 21)
	n (%)	n (%)	n (%)	n (%)	n (%)	n (%)
Age, years median value (range)	73 (42–91)	72 (59–89)	73 (42–91)	75 (48–89)	75 (48–89)	76 (64–86)
**Gleason score**						
6–7	73 (41.5)	18 (51.4)	55 (39.0)	19 (39.6)	13 (41.9)	6 (35.3)
8–10	103 (58.5)	17 (48.6)	86 (61.0)	29 (60.4)	18 (58.1)	11 (64.7)
Unknown/missing	21	5	16	11	7	4
**Metastastic sites**						
No visceral	162 (82.6)	39 (97.5)	123 (78.8)	58 (98.3)	37 (97.4)	21 (100)
Visceral	34 (17.4)	1 (2.5)	33 (21.1)	1 (1.7)	1 (2.6)	0
Unknown	1	0	1	0	0	0
**Lines of previous treatments**						
≤2	122 (61.9)	40 (100)	82 (52.2)	51 (86.4)	37 (97.4)	14 (66.7)
>2	75 (38.1)	0	75 (47.8)	8 (13.6)	1 (2.6)	7 (33.3)
**Previous abi or enza treatment**						
No	147 (74.6)	36 (90.0)	111 (70.7)	54 (91.5)	36 (94.7)	18 (85.7)
Yes	50 (25.4)	4 (10.0)	46 (29.3)	5 (8.5)	2 (5.3)	3 (14.3)
**ECOG**						
0–1	180 (91.4)	39 (97.5)	141 (89.8)	56 (94.9)	35 (92.1)	21 (100)
≥2	17 (8.6)	1 (2.5)	16 (10.2)	3 (5.1)	3 (7.9)	0
***AR*** **copy number**						
Normal	119 (60.4)	29 (72.5)	90 (57.3)	48 (81.4)	32 (84.2)	16 (76.2)
Gain	78 (39.6)	11 (27.5)	67 (42.7)	11 (18.6)	6 (15.8)	5 (23.8)
***AR*** **mutations**						
No	181 (91.9)	38 (95.0)	143 (91.1)	57 (96.6)	37 (97.4)	20 (95.2)
Yes	16 (8.1)	2 (5.0)	14 (8.9)	2 (3.4)	1 (2.6)	1 (4.8)
**CgA**, **ng/mL**						
<120	92 (46.7)	33 (82.5)	59 (37.6)	30 (50.9)	20 (52.6)	10 (47.6)
120–360	66 (33.5)	6 (15.0)	60 (38.2)	18 (30.5)	12 (31.6)	6 (28.6)
>360	39 (19.8)	1 (2.5)	38 (24.2)	11 (18.6)	6 (15.8)	5 (23.8)
Baseline ALP, U/L median value (range)	112 (35–1345)	97 (44–310)	120 (35–1345)	110 (12–321)	110 (12–321)	116 (40–259)
<129	83 (56.5)	20 (66.7)	63 (53.8)	22 (66.7)	16 (69.6)	6 (60.0)
≥129	64 (43.5)	10 (33.3)	54 (46.2)	11 (33.3)	7 (30.4)	4 (40.0)
Unknown	50	10	40	26	15	11
Baseline LDH, U/L median value (range)	186 (67–1808)	156 (67–711)	194 (97–1808)	179 (88–695)	176 (88–675)	199 (98–695)
<225	116 (72.0)	33 (86.8)	83 (67.5)	41 (85.4)	431 (91.2)	10 (71.4)
≥225	45 (28.0)	5 (13.2)	40 (32.5)	7 (14.6)	3 (8.8)	4 (28.6)
Unknown	36	2	34	11	4	7
**Baseline NLR**						
<3	104 (58.1)	20 (55.6)	84 (58.7)	19 (48.7)	14 (50.0)	5 (45.5)
≥3	75 (41.9)	16 (44.4)	59 (41.3)	20 (51.3)	14 (50.0)	6 (54.5)
Unknown/missing	18	4	14	20	10	10
Baseline Hb, g/dL median value (range)	12 (7–15.5)	13.1 (10.2–15.5)	11.8 (7.0–15.1)	12.2 (10.8–101)	12.3 (10.8–101)	12 (11–13)
Baseline PSA, ng/mL median value (range)	41.6 (1.48–4351)	27.45 (1.48–183.3)	51.98 1.48–4351)	20.5 (1.48–4294)	27.02 (1.48–429)	17.39 (3.80–1899)

*Abbreviations*. Abi, abirtaerone; ALP, alkaline phosphatase; *AR*, androgen receptor; CgA, chromogranin A; ECOG, Eastern Cooperative Oncology Group; enza, enzalutamide; Hb, haemoglobin; LDH, lactate dehydrogenase; n, number; NLR, neutrophil to lymphocyte ratio; PLR, platelets to lymphocyte ratio; PSA, prostate-specific antigen.

### Association between baseline cell-free *AR* aberrations and serum CgA

No significant differences were observed in either cohort between cell-free *AR* status and CgA measurements, with the exception of a trend (p = 0.057) of higher CgA level in *AR*-gained chemotherapy-naïve patients of the secondary cohort (Table 2). Using a different cut-off for CgA levels, we observed concordant results showing no correlation between *AR* status and serum CgA concentration (Supplementary Table S1).

**Table 2:** Association between baseline cell-free *AR* aberrations and serum CgA in the Primary and Secondary cohort.

	Primary cohort						Secondary cohort					
	*AR* copy number			*AR* mutations			*AR* copy number			*AR* mutations		
	Normal (n = 119)	Gain (n = 78)	p	No (n = 181)	Yes (n = 16)	p	Normal (n = 48)	Gain (n = 11)	p	No (n = 57)	Yes (n = 2)	p
**Abi/Enza pre-docetaxel**, **n** (**%**)												
CgA <120	25 (86.2)	8 (72.7)		31 (81.6)	2 (100.0)		19 (59.4)	1 (16.7)		20 (54.1)	0 (0)	
120–360	3 (10.3)	3 (27.3)		6 (15.8)	0 (0)		9 (28.1)	3 (50.0)		12 (32.4)	0 (0)	
>360	1 (3.5)	0 (0)	0.542	1 (2.6)	0 (0)	0.532	4 (12.5)	2 (33.3)	0.057	5 (13.5)	1 (100)	0.065
**Abi/Enza post-docetaxel**, **n** (**%**)												
CgA <120	35 (38.9)	24 (35.8)		55 (38.5)	4 (28.6)		7 (43.7)	3 (60.0)		10 (50.0)	0 (0)	
120–360	39 (43.3)	21 (31.3)		55 (38.5)	5 (35.7)		4 (25.0)	2 (40.0)		5 (25.0)	1 (100)	
>360	16 (17.8)	22 (32.9)	0.148	33 (23.1)	5 (35.7)	0.300	5 (31.3)	0 (0)	0.264	5 (25.0)	0 (0)	0.769

*Abbreviations*. Abi, abiraterone; AR, androgen receptor; CgA, chromogranin A; enza, enzalutamide; n, number; UNV, upper normal value.

### Combination of *AR* status with CgA levels and PSA response

Median baseline PSA was 44.5 ng/mL (range, 1.48–4351), and our results did not reveal a significant difference between PSA and CgA levels (Supplementary Table S2) (as commonly shown in NEPC)^24,30^.

No association was seen between PSA response and CgA levels according to cell-free *AR* copy number (CN) in the primary and secondary cohort (Supplementary Table S3).

### Combination of *AR* status with CgA levels and survival

The median follow-up of patients from the primary and secondary cohort was 32.4 months (range, 1–73) and 17.6 months (range, 1–49), respectively.

We did not evaluate the impact of *AR* mutations on treatment outcome because cell-free *AR* mutations were uncommon, especially in chemotherapy-naïve patients, as shown in previous studies^10,12,13^, and the clinical relevance of rarer mutations is uncertain. Our droplet digital PCR (ddPCR) assay detected point mutations present in at least 2% of plasma DNA.

**Figure 1:**
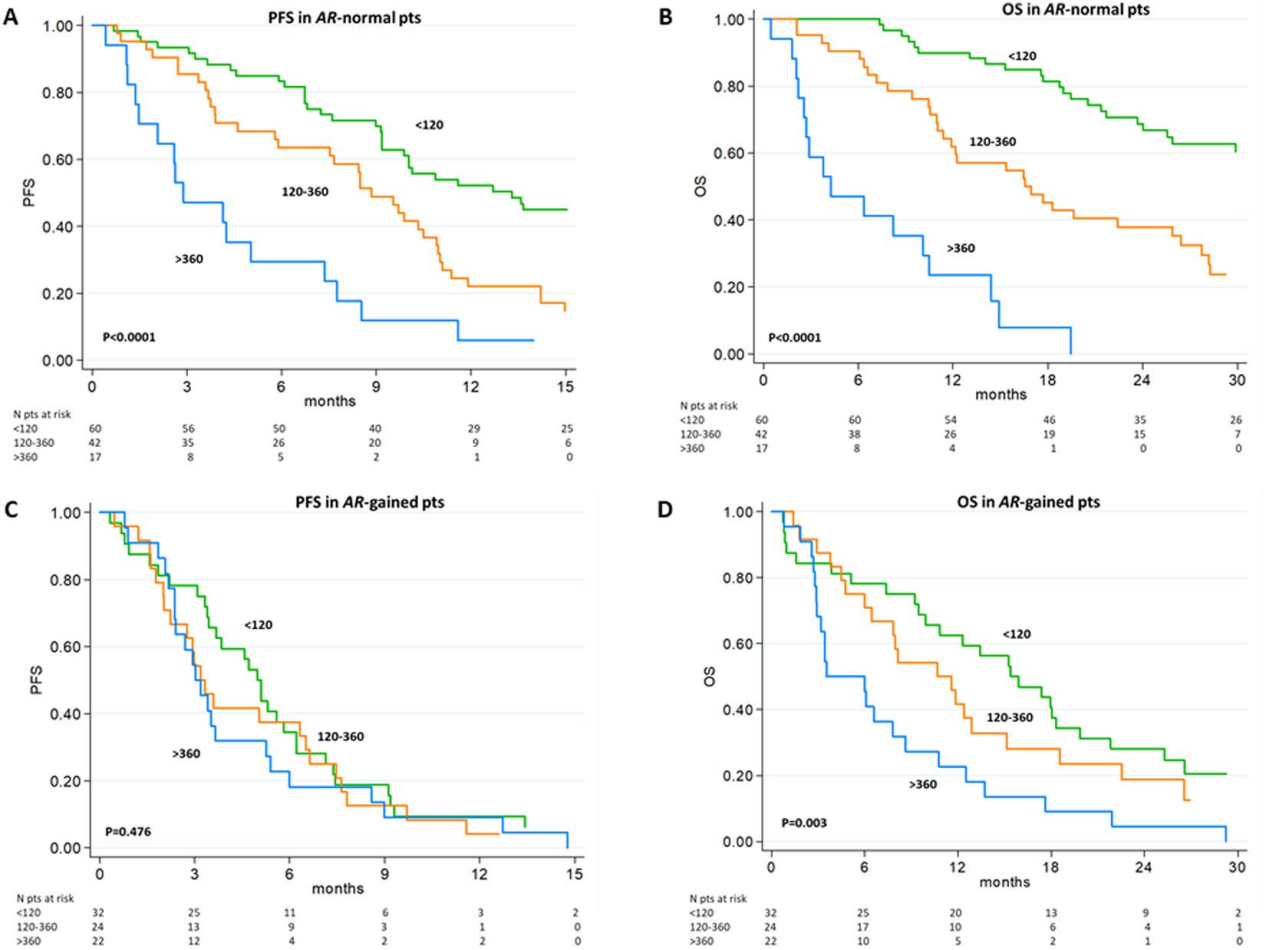
Survival in patients treated with abiraterone or enzalutamide by *AR* status and CgA levels in the Primary cohort. PFS (**A**) and OS (**B**) in *AR*-normal and PFS (**C**) and OS (**D**) in *AR*-gained patients according to three different CgA level groups. The blue line is for patients with serum CgA <120 ng/mL, the orange line for CgA between 120 and 360 ng/mL, and the green line for CgA level >360 ng/mL.

**Figure 2:**
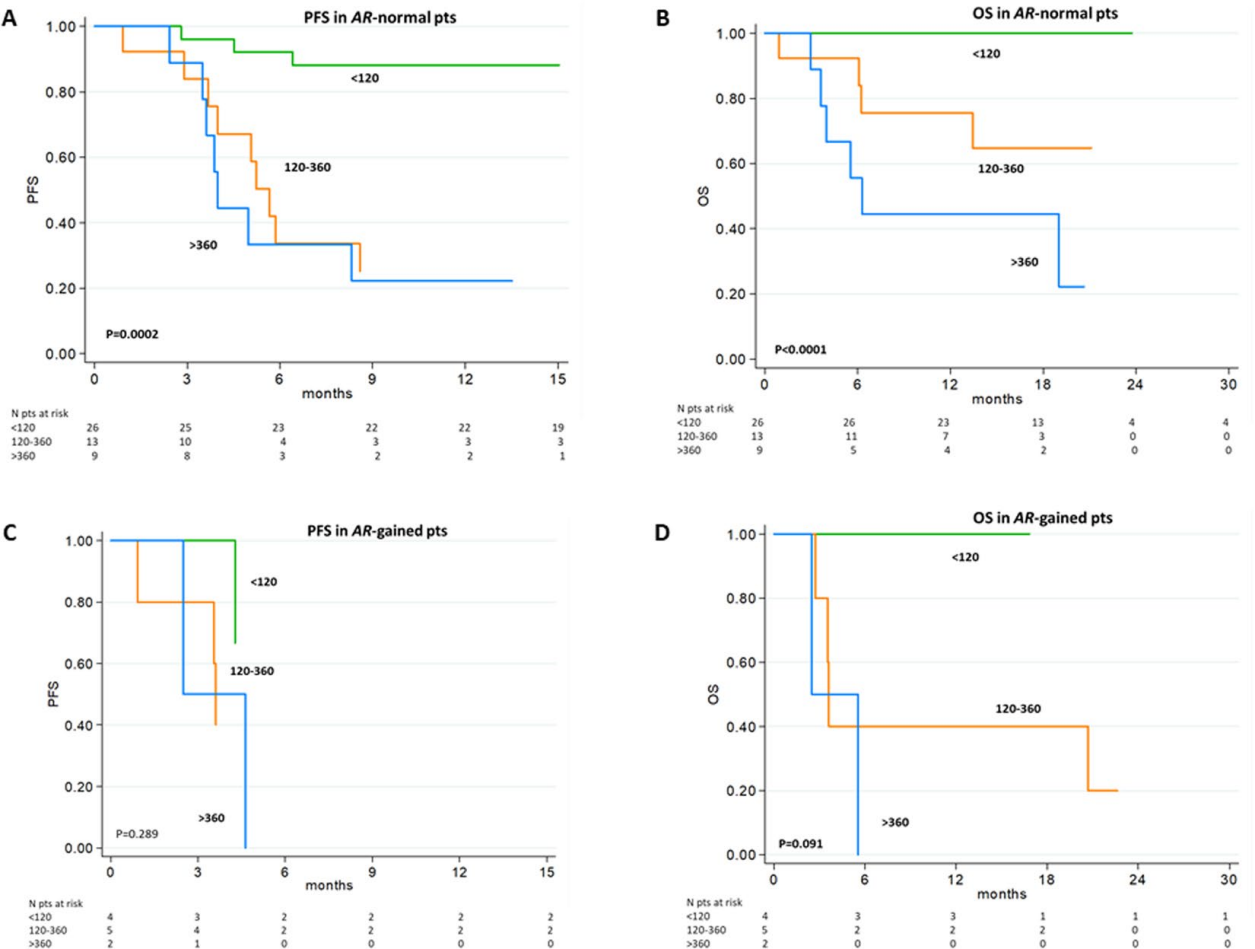
Survival in patients treated with abiraterone or enzalutamide by *AR* status and CgA levels in the Secondary cohort. PFS (**A**) and OS (**B**) in *AR*-normal and PFS (**C**) and OS (**D**) in *AR*-gained patients according to three different CgA level groups. The blue line is for patients with serum CGA <120 ng/mL, the orange line for CGA between 120 and 360 ng/mL, and the green line for CGA level >360 ng/mL.

In both cohorts, we observed a significant association between worse progression-free/overall survival (PFS/OS) and elevated CgA levels in *AR*-normal patients treated with abiraterone or enzalutamide, more evident in post-docetaxel setting for the primary cohort or in pre-docetaxel for the secondary cohort (Figs 1 and 2; Supplementary Tables S4 and S5).

Multivariable analysis revealed *AR* gain and higher CgA and lactate dehydrogenase (LDH) levels as an independent predictors of PFS [hazard ratio (HR) 2.16, 95% confidence interval (CI) 1.50–3.12, p < 0.0001, HR 1.73, 95% CI 1.06–2.84, p = 0.026, and HR 2.13, 95% CI 1.45–3.13, p = 0.0001, respectively) and OS (HR 1.72, 95% CI 1.15–2.57, p = 0.008, HR 3.63, 95% CI 2.13–6.20, p < 0.0001, and HR 2.31, 95% CI 1.54–3.48, p < 0.0001, respectively) (Supplementary Table S6). In the secondary cohort, higher CgA concentration was confirmed as independent predictors of PFS and OS (HR 4.16, 95% CI 1.51–11.49, p = 0.006 and HR 62.97, 95% CI 5.08–781.12, p = 0.001, respectively), together with *AR* gain and elevated LDH level.

In overall patient population, interactions between treatment (abiraterone versus enzalutamide) and *AR* status examined in the Cox models were not significant (p = 0.131 for PFS and p = 0.598 for OS).

## Discussion

The growing use of AR-directed agents and a higher number of biopsies performed not only at diagnosis, but also at different time points during the course of prostate cancer are leading to an increased detection of NEPCs, especially treatment-related forms. However, the challenge to repeat biopsies before starting new treatment for CRPC is not always feasible and in such cases only elevated serum neuroendocrine markers can facilitate the diagnosis of these particular forms of prostate cancer. Our study, which excluded patients with NEPC or small cell carcinoma histology and aimed to evaluate the clinical impact of determining circulating NED biomarkers in metastatic CRPC patients before a systemic treatment.

Assuming that CgA is a reliable prognostic marker^29,30^, we divided the patients into different subgroups based on pre-therapy CgA levels. Patients treated with abiraterone/enzalutamide post-docetaxel underwent at least 2 different treatments and showed more elevated serum CgA level than chemotherapy-naïve cases indicating that NED is often secondary to androgen deprivation therapy. In addition, higher levels of CgA were observed in patients with visceral metastases, especially in the liver, and those with increased LDH values and NLR ≥3. This would seem to indicate that association of NED is associated with extent of disease and inflammation in these mixed tumor forms, as in NEPC^24,31–33^.

The primary histology of cases included in this work excluded a neuroendocrine component. However, our adenocarcinoma cases had hybrid features with both *AR* and neuroendocrine markers present and high PSA levels. It is possible that these “mixed forms” represent an earlier step of NEPC development nearing the end of an *AR*-dependent state with a paucity of somatic alterations involving the *AR* gene^24^. Although a pathological review of all included cases would have been useful, especially during treatment, at progression and/or during follow-up; the majority of patients did not undergo further biopsy.

The present study confirmed the prognostic impact of higher CgA levels on PFS and OS and the lack of an association between CgA concentration and PSA response, as reported in other works^28–30,34^. We used CgA levels > 3-fold the UNV as the best cut-off to identify a poorer prognostic group, whereas CgA levels > 5-fold the UNV^35,36^ did not provide robust evidence, probably because of the number of patients in the different groups was not well-balanced in terms of *AR* status.

Interestingly, we correlated, for the first time, survival data with the presence of cell-free *AR* aberrations and serum CgA levels, suggesting that the presence of elevated CgA concentration could identify CRPC patients at high-risk of developing NEPC and resistance to AR-signalling inhibitors.

Our multivariable analysis confirmed baseline CgA as an independent predictor of survival and further highlighted the usefulness of *AR* CN^12–17^ and LDH^37^ as prognostic markers.

The limitations of this study were its retrospective design, small number of patients included in the different CgA prognostic groups and high number of previous treatments that influenced the homogeneity of results. Moreover, the characteristics of the 2 patient cohorts^38^ differed slightly but with an uncertain clinical impact (the secondary cohort had a higher number of chemotherapy-naïve patients and a shorter median follow-up period than the primary cohort). A major limitation of this study was the lack of data on *AR* splice variants^19^, as a potential alternative mechanism of therapeutic resistance.

Genome wide DNA sequencing could also be used to study other molecular mechanisms that may lead to the progression of CRPC to NEPC, an effort to define a molecular phenotype of mixed forms between prostate adenocarcinoma and NEPC in association with CgA levels. These include loss of tumor suppressors, such as RB1 and p53^24,39,40^, and amplification of MYCN^24^. In addition, the study of non-genomic factors such as PEG10^41^, splicing factors like SSRM4^42,43^, and AKT activation overexpression of the master neural transcription factor BRN2^44^ could contribute to better determining these tumors from a genomic point of view.

Given that germline mutations of DNA repair–deficient prostate cancer have been associated with resistance to abiraterone and enzalutamide^38^ and that these alterations were recently linked with NED molecular pathways sharing common platinum-sensitivity^45^, mixed forms between prostate adenocarcinoma and NEPC could be also characterized on the basis of a deficit in DNA damage repair, especially in aggressive CRPC variants with *AR* CN normal.

In conclusion, the evaluation of serum CgA could be key in improving the management of CRPC patients displaying a mixed tumor form between adenocarcinoma and NEPC and treated with standard therapies. Given that these hubris tumors may retain active AR expression and/or signalling, 2 therapeutic options for this patient subgroup could be platinum-based chemotherapy. A combined therapeutic strategy including hormonal drugs and other chemotherapeutic agents or targeted therapies (such as aurora kinase inhibitors^24^ or BRN2^44^) is probably needed to overcome resistance related to the onset of CgA-expressing clones.

There is now sufficient evidence to warrant carrying out clinical trials that prospectively select treatment on the basis of baseline NED markers such as CgA and molecular alterations that drive progression towards NED phenotype. Further studies may provide a robust evidence for a change of clinical care for this distinct tumor subclass.

## Methods

### Patients

We collected pre-treatment plasma samples of 256 patients enrolled in 2 biomarkers studies approved by the Institutional Review Board of Istituto Scientifico Romagnolo per lo Studio e la Cura dei Tumori (IRST) IRCCS, Meldola, Italy. We selected 197 CRPC patients of the primary cohort between March 2013 and July 2015, and 59 patients of the secondary cohort between March 2015 and March 2017. All patients had histology of prostate adenocarcinoma without NED and a progressive disease despite “castration levels” of serum testosterone (<50 ng/dL), ongoing LHRH analogue treatment or prior surgical castration. Additional eligibility criteria included an Eastern Cooperative Oncology Group (ECOG) performance status 0–2, adequate cardiac, renal, hepatic and bone marrow function. Exclusion criteria were renal insufficiency and/or concomitant therapy with proton pump inhibitors, which could influence the CgA levels^46^.

Treatment consisted of anti-AR therapies including abiraterone 1 g once a day and prednisone 5 mg twice daily, or enzalutamide 160 mg once a day, in either as pre- or post-chemotherapy setting. The choice of therapy was at the discretion of the treating physician. Therapies were administered continuously until there was evidence of disease progression or unacceptable toxicity. The studies were conducted in accordance with the Declaration of Helsinki and the Good Clinical Practice guidelines of the International Conference of Harmonization. Written informed consent was obtained from all patients.

### Procedures

Serum CgA levels were measured in duplicate using a two-sided “sandwich” technique with two selected antibodies that bind to different epitopes of human CgA (Epitope Diagnostics, Inc - EDI Human Chromogranin A ELISA Kit, San Diego, CA), in accordance with the manufacturer’s instructions. The sensitivity was 2 mg/L. The inter-assay coefficients of variation of CgA assay were 7.3% and 3.1%. The normal range reported by the kit for CgA was 0–120 ng/mL.

All patients underwent a history evaluation and physical examination, and blood tests including complete blood cell count, serum PSA, alkaline phosphatase (ALP), and LDH the week before each treatment cycle. PSA and blood tests were performed on a monthly basis. Serum CgA levels were determined at baseline and upon disease progression. Radiographic evaluation was performed by computed tomography and bone scan at the time of screening and every 12 weeks thereafter. Response was evaluated according to Prostate Cancer Working Group (PCWG2) guidelines^47^ and soft tissue disease was assessed using Response Evaluation Criteria in Solid Tumors (RECIST) version 1.1. Peripheral blood samples were collected within 30 days of the start of treatment and plasma aliquots were stored at −80 °C. Circulating DNA was extracted from one to two mL of plasma with the QIAamp Circulating Nucleic Acid Kit (Qiagen). Total extracted plasma DNA was quantified by 2 methods for a major accuracy: spectrophotometric evaluation (NanoDrop® ND-1000, Celbio, Milan, Italy) and Quant-iT high sensitivity PicoGreen double-stranded DNA Assay Kit (Invitrogen) for maximum accuracy. AR aberrations [CN and somatic point mutations: 2105 T > A (p.L702H) and 2632 A > G (p.T878A) with a limit of detection of 1–2% using an input of 2 to 4 ng of DNA] were detected by multiplex ddPCR on a QX200 ddPCR system (Bio-Rad)^12–14^.

For *AR* CN analyses, we used the AR gene and at least two different reference genes: *RNaseP*, *NSUN3*, *ElF2C1*, and *AP3B1* and *ZXDB* at Xp11.21 as a control gene.

### Statistical analysis

The primary endpoint of this study was the association between circulating *AR* aberrations and CgA levels. The secondary endpoints were PFS/OS and PSA response rate (RR) (>50% PSA decline after 12 weeks of treatment) stratified by circulating *AR* status and CgA level.

Data were summarized by frequency for categorical variables and by median and range for continuous variables. The Wilcoxon rank sum test was performed to compare continuous variables and Chi-Square or Fisher’s exact test were performed to compare categorical variables, as appropriate. The cut-offs for CgA in response to therapies were determined through ROC curve analysis, as previously reported^28,29^. Consequently, all patients were subdivided into three groups: (1) normal serum CgA level < 120 ng/mL, (2) < 3-fold the UNV 120–360 ng/mL, and (3) > 3-fold the UNV > 360 ng/mL). In the exploratory sub-analysis, we also used 5-fold the UNV to evaluate a potentially more stringent cut-off.

PSA response was considered as a ≥50% decline after a minimum of 12 weeks treatment confirmed by a second PSA test after a minimum of four weeks. PFS was calculated from the start of each therapy until the first date of progression, death from any cause, or last tumor evaluation. OS was calculated from the start of each therapy until death or last follow-up. Survival curves were estimated by the Kaplan-Meier method and were compared using the log-rank test. Univariate and multivariate Cox regression models were used to investigate potential predictors of PFS and OS and to estimate HR and their 95% CI. For these analyses, we included different clinically relevant factors as covariates for both cohorts (age, cell-free *AR* CN, CgA level, chemotherapy status, number of previous treatment lines, Gleason score, site of metastasis, and baseline serum LDH and PSA levels). We also conducted landmark analysis to reduce the potential for time-dependent confounding in treatment by assessing the impact of changes in serum CgA level from baseline to progression on survival outcome.

All P-values were two-sided and a p < 0.05 was considered as statistically significant. Statistical analyses were performed with SAS software version 9.4 (SAS Institute, Cary, NC, USA).

## Electronic supplementary material

Dataset 1
