# Supplementary material for: Plasma androgen receptor and serum chromogranin A in advanced prostate cancer
Source: Sci Rep. 2018 Oct 18;8:15442. doi: 10.1038/s41598-018-33774-4 (PMC6194135; doi:10.1038/s41598-018-33774-4)
Supplement: Supplementary file 1 — Dataset 1 [file 41598_2018_33774_MOESM1_ESM.doc]

**Supplementary data**

**Plasma androgen receptor and serum chromogranin A in advanced prostate cancer**

**Running Title:** Plasma AR and chromogranin A

Vincenza Conteduca^1,2^, Emanuela Scarpi^3^, Samanta Salvi^4^, Valentina Casadio^4^, Cristian Lolli^1^, Giorgia Gurioli^4^, Giuseppe Schepisi^1^, Daniel Wetterskog^2^, Alberto Farolfi^1^, Cecilia Menna^1^, Delia De Lisi^5^, Salvatore Luca Burgio^1^, Himisha Beltran^6^, Gerhardt Attard^2,7^, Ugo De Giorgi^1^

^1^Department of Medical Oncology, Istituto Scientifico Romagnolo per lo Studio e la Cura dei Tumori (IRST) IRCCS, via Maroncelli 40, 47014 Meldola, Italy.

^2^The Institute of Cancer Research and the Royal Marsden, 15 Cotswold Road, Sutton, Surrey SM2 5NG, UK.

^3^Unit of Biostatistics and Clinical Trials, Istituto Scientifico Romagnolo per lo Studio e la Cura dei Tumori (IRST) IRCCS, via Maroncelli 40, 47014 Meldola, Italy.

^4^Biosciences Laboratory, Istituto Scientifico Romagnolo per lo Studio e la Cura dei Tumori (IRST) IRCCS, via Maroncelli 40, 47014 Meldola, Italy.

^5^Medical Oncology Department, Campus Bio-Medico University, Via Alvaro del Portillo 200, 00128 Rome, Italy.

^6^Division of Medical Oncology, Weill Cornell Medicine, New York, NY 10021, USA.

^7^Academic Urology Unit, The Royal Marsden NHS Foundation Trust, London, UK.

**Table S1. Association between baseline cell-free *AR* aberrations and serum CgA (cut-off x 5 UNV) in the Primary and Secondary cohort**

|  | **Primary cohort** | | | | | | **Secondary cohort** | | | | | |
| --- | --- | --- | --- | --- | --- | --- | --- | --- | --- | --- | --- | --- |
|  | ***AR* copy number** | | | ***AR* mutations** | | | ***AR* copy number** | | | ***AR* mutations** | | |
|  | **Normal (n=119)** | **Gain (n=78)** | **p** | **No (n=181)** | **Yes (n=16)** | **p** | **Normal (n=48)** | **Gain (n=11)** | **p** | **No (n=57)** | **Yes (n=2)** | **p** |
| ***Abi/Enza pre-docetaxel, n (%)*** |  |  |  |  |  |  |  |  |  |  |  |  |
| CgA <120 | 25 (86.2) | 8 (72.7) |  | 31 (81.6) | 2 (100.0) |  | 19 (59.4) | 1 (16.7) |  | 20 (54.1) | 0 (0) |  |
| 120-600 | 3 (10.3) | 3 (27.3) |  | 6 (15.8) | 0 (0) |  | 13 (40.6) | 5 (83.3) |  | 17 (45.9) | 1 (100) |  |
| >600 | 1 (3.5) | 0 (0) | 0.542 | 1 (2.6) | 0 (0) | 0.532 | 0 (0) | 0 (0) | ne | 0 (0) | 0 (0) | ne |
| ***Abi/Enza post-docetaxel, n (%)*** |  |  |  |  |  |  |  |  |  |  |  |  |
| CgA <120 | 35 (38.9) | 24 (35.8) |  | 55 (38.5) | 4 (28.6) |  | 7 (43.7) | 3 (60.0) |  | 10 (50.0) | 0 (0) |  |
| 120-600 | 49 (54.4) | 32 (47.8) |  | 74 (51.7) | 7 (50.0) |  | 8 (50.0) | 2 (40.0) |  | 9 (45.0) | 1 (100) |  |
| >600 | 6 (6.7) | 11 (16.4) | 0.218 | 14 (9.8) | 3 (21.4) | 0.233 | 1 (6.3) | 0 (0) | 0.462 | 1 (5.0) | 0 (0) | 0.462 |

Abbreviations. Abi, abiraterone; AR, androgen receptor; CgA, chromogranin A; enza, enzalutamide; n, number; UNV, upper normal value.

**Table S2. Baseline PSA values according to different CgA groups in the Primary and Secondary cohort**

|  | **Primary Cohort** | |  | | | | |  | **Secondary Cohort** | | | | | | | |  |
| --- | --- | --- | --- | --- | --- | --- | --- | --- | --- | --- | --- | --- | --- | --- | --- | --- | --- |
|  | **PSA <5ng/mL**  **n (%)** | **PSA ≥5 ng/mL**  **n (%)** | | | | | **p** | | | | | **PSA <5ng/mL**  **n (%)** | | **PSA ≥5 ng/mL**  **n (%)** | | **p** | |
| **Overall** |  |  | |  | | | | | | |  | |  | |  | |  |
| **CgA** |  |  | |  | | | | | | |  | |  | |  | |  |
| ≤120 | 16 (69.6) | 76 (43.7) | |  | | | | | | | 10 (100) | | 20 (40.8) | |  | |  |
| 121-360 | 4 (17.4) | 62 (35.6) | | |  | | | | | | 0 | | 18 (36.7) | |  | |  |
| >360 | 3 (13.0) | 36 (20.7) | | | 0.050 | | | | | | 0 | | 11 (22.5) | | 0.002 | |  |
|  |  |  | | |  | | | | | |  | |  | |  | |  |
| **CgA** |  |  | | |  | | | | | |  | |  | |  | |  |
| ≤120 | 7 (100) | 26 (78.8) | | |  | | | | | | 9 (100) | | 11 (37.9) | |  | |  |
| 121-360 | 0 | 6 (18.2) | | |  | | | | | | 0 | | 12 (41.4) | |  | |  |
| >360 | 0 | 1 (3.0) | | | 0.209 | | | | | | 0 | | 6 (20.7) | | 0.004 | |  |
|  |  |  | | |  | | | | | |  | |  | |  | |  |
| **CgA** |  |  | | |  | | | | | |  | |  | |  | |  |
| ≤120 | 9 (56.2) | 50 (35.5) | | |  | | | | | | 1 (100) | | 9 (45.0) | |  | |  |
| 121-360 | 4 (25.0) | 56 (39.7) | | |  | | | | | | 0 | | 6 (30.0) | |  | |  |
| >360 | 3 (18.8) | 35 (24.8) | | | | 0.190 | | | | 0 | | | 5 (25.0) | | 0.347 | |  |

*Abbreviations.* Abi, abirateraterone; CgA, chromogranin A; Enza, enzalutamide; n, number; PSA, prostate specific antigen

**Table S3. PSA response according to cell-free *AR* copy number and CgA level in the Primary and Secondary cohort**

|  | **PSA RR in Primary cohort** | | | | | | **PSA RR in Secondary cohort** | | | | | |
| --- | --- | --- | --- | --- | --- | --- | --- | --- | --- | --- | --- | --- |
|  | ***AR* normal** | | | ***AR* gain** | | | ***AR* normal** | | | ***AR* gain** | | |
|  | **Yes n (%)** | **No**  **n (%)** | **p** | **Yes n (%)** | **No n (%)** | **p** | **Yes n (%)** | **No n (%)** | **p** | **Yes n (%)** | **No n (%)** | **p** |
| ***Abi/Enza pre-docetaxel, n (%)*** |  |  |  |  |  |  |  |  |  |  |  |  |
| CgA <120 | 20 (91.0) | 5 (71.4) |  | 5 (71.4) | 3 (75.0) |  | 14 (58.3) | 5 (62.5) |  | 1 (50.0) | 0 |  |
| 120-360 | 1 (4.5) | 2 (28.6) |  | 2 (28.6) | 1 (25.0) |  | 6 (25.0) | 3 (37.5) |  | 0 | 3 (75.0) |  |
| >360 | 1 (4.5) | 0 | 0.462 | 0 | 0 | ne | 4 (16.7) | 0 | 0.477 | 1 (50.0) | 1 (25.0) | 0.701 |
| ***Abi/Enza post-docetaxel, n (%)*** |  |  |  |  |  |  |  |  |  |  |  |  |
| CgA <120 | 24 (57.2) | 11 (22.9) |  | 4 (26.7) | 20 (38.5) |  | 5 (55.6) | 2 (28.6) |  | 1 (33.3) | 2 (100) |  |
| 120-360 | 14 (33.3) | 25 (52.1) |  | 6 (40.0) | 15 (28.8) |  | 2 (22.2) | 2 (28.6) |  | 2 (66.7) | 0 |  |
| >360 | 4 (9.5) | 12 (25.0) | 0.001 | 5 (33.3) | 17 (32.7) | 0.611 | 2 (22.2) | 3 (42.8) | 0.286 | 0 | 0 | ne |

*Abbreviations.* Abi, abiraterone; *AR*, androgen receptor; CgA, chromogranin A; enza, enzalutamide; n, number; ne, not estimable; PSA, prostate specific antigen; RR, response rate.

Table S4. Progression-free and overall survival in the Primary cohort

|  |  | **PFS** | | | | | **OS** | | | | |
| --- | --- | --- | --- | --- | --- | --- | --- | --- | --- | --- | --- |
|  | **N. pts** | **N. events** | **Median PFS (months)**  **(95% CI)** | **p** | **HR**  **(95% CI)** | **p** | **N. events** | **Median OS (months)**  **(95% CI)** | **p** | **HR**  **(95% CI)** | **p** |
| ***Total Abi/Enza pre- and post-docetaxel*** | | | | | | | | | | | |
| **Overall**  ***AR* Normal** | 197 | 176 | 6.7 (5.3-7.8) | - | - | - | 152 | 16.6 (12.9-19.0) | - | - | - |
| CgA <120 | 60 | 45 | 13.3 (9.2-17.7) |  | 1.00 |  | 32 | 35.1 (25.6-45.8) |  | 1.00 |  |
| 120-360 | 42 | 40 | 8.8 (5.8-10.9) |  | 2.05 (1.33-3.18) |  | 36 | 16.8 (11.4-25.9) |  | 2.42 (1.49-3.91) |  |
| >360 | 17 | 16 | 2.9 (1.4-7.4) | <0.0001 | 4.88 (2.66-8.95) | <0.0001 | 16 | 4.3 (2.2-10.5) | <0.0001 | 10.68 (5.47-20.84) | <0.0001 |
|  |  |  |  |  |  |  |  |  |  |  |  |
| ***AR* Gain** |  |  |  |  |  |  |  |  |  |  |  |
| CgA <120 | 32 | 30 | 5.0 (3.4-6.2) |  | 1.00 |  | 26 | 15.6 (9.5-19.8) |  | 1.00 |  |
| 120-360 | 24 | 23 | 3.3 (2.0-6.5) |  | 1.24 (0.72-2.16) |  | 20 | 11.1 (6.0-15.1) |  | 1.33 (0.74-2.40) |  |
| >360 | 22 | 22 | 3.1 (3.1-5.3) | 0.476 | 1.39 (0.80-2.42) | 0.480 | 22 | 4.8 (2.9-8.6) | 0.003 | 2.62 (1.46-4.70) | 0.004 |
|  |  |  |  |  |  |  |  |  |  |  |  |
| ***Abi/Enza pre-docetaxel*** | | | | | | | | | | | |
| **Overall**  ***AR* Normal** | 40 | 25 | 16.1 (9.1-24.4) | - | - | - | 12 | nr | - | - | - |
| CgA <120 | 25 | 14 | 21.2 (13.6-nr) |  | 1.00 |  | 2 | nr |  | 1.00 |  |
| 120-360 | 3 | 2 | 5.9 (3.7-nr) |  | 2.88 (0.63-13.11) |  | 1 | nr |  | 5.36 (0.48-59.63) |  |
| >360 | 1 | 0 | nr | 0.287 | - | 0.394 | 0 | nr | 0.281 | - | 0.394 |
|  |  |  |  |  |  |  |  |  |  |  |  |
| ***AR* Gain** |  |  |  |  |  |  |  |  |  |  |  |
| CgA <120 | 8 | 7 | 5.2 (3.5-9.1) |  | 1.00 |  | 7 | 18.1 (5.1-26.6) |  | 1.00 |  |
| 120-360 | 3 | 2 | 11.6 (2.0-nr) |  | 0.50 (0.10-2.60) |  | 2 | 12.4 (11.6-nr) |  | 1.46 (0.24-8.82) |  |
| >360 | 0 | 0 | - | 0.407 | - | 0.414 | 0 | - | 0.675 | - | 0.677 |
|  |  |  |  |  |  |  |  |  |  |  |  |
| ***Abi/Enza post-docetaxel*** | | | | | | | | | | | |
| **Overall**  ***AR* Normal** | 157 | 151 | 5.8 (3.9-6.8) | - | - | - | 140 | 13.4 (10.7-16.6) | - | - | - |
| CgA <120 | 35 | 31 | 10.0 (6.7-12.7) |  | 1.00 |  | 30 | 24.0 (18.7-31.7) |  | 1.00 |  |
| 120-360 | 39 | 38 | 9.5 (5.8-10.9) |  | 1.41 (0.87-2.29) |  | 35 | 16.6 (11.0-25.9) |  | 1.50 (0.92-2.45) |  |
| >360 | 16 | 16 | 2.8 (1.4-5.0) | <0.0001 | 4.33 (2.26-8.29) | <0.0001 | 16 | 4.0 (2.2-10.1) | <0.0001 | 7.61 (3.86-15.02) | <0.0001 |
|  |  |  |  |  |  |  |  |  |  |  |  |
| ***AR* Gain** |  |  |  |  |  |  |  |  |  |  |  |
| CgA <120 | 24 | 23 | 4.8 (2.2-6.2) |  | 1.00 |  | 19 | 15.3 (9.2-21.8) |  | 1.00 |  |
| 120-360 | 21 | 21 | 3.2 (2.0-6.3) |  | 1.36 (0.75-2.48) |  | 18 | 8.1 (4.8-15.1) |  | 1.35 (0.70-2.57) |  |
| >360 | 22 | 22 | 3.1 (2.4-5.3) | 0.572 | 1.25 (0.69-2.24) | 0.576 | 22 | 4.8 (2.9-8.6) | 0.011 | 2.52 (1.34-4.73) | 0.013 |

*Abbreviations.* Abi, abiraterone; *AR,* androgen receptor; CgA, chromogranin A; CI, confidence interval; Enza, enzalutamide; HR, hazard ratio; N, number; nr=not reached; PFS, progression-free survival; pts, patients; OS, overall survival.

Table S5. Progression-free and overall survival in the Secondary Cohort

|  |  | **PFS** | | | | | **OS** | | | | |
| --- | --- | --- | --- | --- | --- | --- | --- | --- | --- | --- | --- |
|  | **N. pts** | **N. events** | **Median PFS (months)**  **(95% CI)** | **p** | **HR**  **(95% CI)** | **p** | **N. events** | **Median OS (months)**  **(95% CI)** | **p** | **HR**  **(95% CI)** | **p** |
| ***Total Abi/Enza pre- and post-docetaxel*** | | | | | | | | | | | |
| **Overall**  ***AR* Normal** | 59 | 31 | 16.4 (5.7-39.7) | - | - | - |  |  | - | - | - |
| CgA <120 | 26 | 7 | 22.2 (20.6-nr) |  | 1.00 |  | 0 | nr |  | 1.00 |  |
| 120-360 | 13 | 9 | 5.7 (2.9-nr) |  | 5.53 (1.92-15.90) |  | 4 | nr |  | ne |  |
| >360 | 9 | 7 | 4.0 (2.4-nr) | 0.0002 | 6.65 (2.19-20.19) | 0.001 | 6 | 6.3 (3.0-nr) | <0.0001 | ne | ne |
|  |  |  |  |  |  |  |  |  |  |  |  |
| ***AR* Gain** |  |  |  |  |  |  |  |  |  |  |  |
| CgA <120 | 4 | 2 | 39.7 (4.3-39.7) |  | 1.00 |  | 0 | nr |  | 1.00 |  |
| 120-360 | 5 | 4 | 3.6 (0.9-nr) |  | 3.84 (0.43-34.52) |  | 4 | 3.6 (2.7-nr) |  | ne |  |
| >360 | 2 | 2 | 3.6 (2.5-4.6) | 0.289 | 5.92 (0.51-68.61) | 0.155 | 2 | 4.0 (2.5-5.6) | 0.091 | ne | ne |
|  |  |  |  |  |  |  |  |  |  |  |  |
| ***Abi/Enza pre-docetaxel*** | | | | | | | | | | | |
| **Overall**  ***AR* Normal** | 38 | 19 | 20.6 (5.9-22.2) | - | - | - | 11 | nr | - | - | - |
| CgA <120 | 19 | 5 | 20.6 (20.6-22.2) |  | 1.00 |  | 0 | nr |  | 1.00 |  |
| 120-360 | 9 | 6 | 5.7 (0.9-nr) |  | 5.38 (1.48-19.51) |  | 3 | nr |  | ne |  |
| >360 | 4 | 3 | 4.2 (2.4-nr) | 0.006 | 6.21 (1.29-29.96) | 0.018 | 3 | 12.3 (3.0-nr) | 0.002 | ne | ne |
|  |  |  |  |  |  |  |  |  |  |  |  |
| ***AR* Gain** |  |  |  |  |  |  |  |  |  |  |  |
| CgA <120 | 1 | 0 | nr |  | 1.00 |  | 0 | nr |  | 1.00 |  |
| 120-360 | 3 | 3 | 3.6 (3.6-15.7) |  | ne |  | 3 | 3.6 (3.6-21.0) |  | ne |  |
| >360 | 2 | 2 | 3.6 (2.5-4.6) | 0.285 | ne | ne | 2 | 4.0 (2.5-5.6) | 0.434 | ne | ne |
|  |  |  |  |  |  |  |  |  |  |  |  |
| ***Abi/Enza post-docetaxel*** | | | | | | | | | | | |
| **Overall**  ***AR* Normal** | 21 | 12 | 8.3 (3.9-39.7) | - | - | - | 5 | nr | - | - | - |
| CgA <120 | 7 | 2 | nr |  | 1.00 |  | 0 | nr |  | 1.00 |  |
| 120-360 | 4 | 3 | 5.1 (2.9-8.6) |  | 4.71 (0.78-28.53) |  | 1 | 13.5 (-) |  | ne |  |
| >360 | 5 | 4 | 4.0 (3.6-nr) | 0.140 | 4.12 (0.73-23.09) | 0.189 | 3 | 6.3 )3.6-nr) | 0.068 | ne | ne |
|  |  |  |  |  |  |  |  |  |  |  |  |
| ***AR* Gain** |  |  |  |  |  |  |  |  |  |  |  |
| CgA <120 | 3 | 2 | 22.0 (4.3-39.7) |  | 1.00 |  | 0 | nr |  | 1.00 |  |
| 120-360 | 2 | 1 | nr |  | 1.73 (0.11-27.89) |  | 1 | nr |  | ne |  |
| >360 | 0 | 0 | - | 0.695 | - | 0.698 | 0 | - | 0.317 | - | ne |

*Abbreviations.* Abi, abiraterone; *AR,* androgen receptor; CgA, chromogranin A; CI, confidence interval; Enza, enzalutamide; HR, hazard ratio; N, number; ne=not estimable; PFS, progression-free survival; pts, patients; OS, overall survival; nr=not reached
